# A stabilized enrichment method for rosemary diterpenoids and their therapeutic potential in diabetic kidney disease

**DOI:** 10.1186/s13020-026-01404-1

**Published:** 2026-05-06

**Authors:** Jiali Wei, Qian Xiao, Hua Yang, Fei Li, Ping Li

**Affiliations:** State Key Laboratory of Natural Medicines, China Pharmaceutical University, Nanjing, 211198 China

**Keywords:** *Rosmarinus officinalis* L., Chemical profiling, Diterpenoids, Enrichment, Anti-diabetic kidney disease effect

## Abstract

**Background:**

As a traditional medicinal herb, rosemary leaves have shown potential for the treatment for diabetic kidney disease (DKD). However, the bioactive compounds responsible for its anti-DKD effects remain unidentified, and the instability of its primary phytochemicals limits its practical application.

**Methods:**

This study investigated the active components of rosemary leaves and characterized their degradation pathways using UPLC-Q/TOF–MS/MS. Four solvent-extracted fractions were evaluated using an in vitro DKD model to identify the major anti-DKD constituents. A targeted enrichment method was then developed to isolate these bioactive compounds. Finally, a DKD model was established in C57BL/6J mice using high-fat diet combined with streptozotocin injection to evaluate the therapeutic effects of the enriched extracts. The pharmacokinetic profiles of the major components of TD were further investigated.

**Results:**

38 chemical constituents were characterized with diterpenoids were identified as the primary anti-DKD constituents by in vitro DKD model. Considering their pronounced susceptibility to oxidative degradation, a novel enrichment strategy for total diterpenoids (TD) was developed based on water and ethanol. Vitamin C served as a stabilizer to improve TD stability, leading to a TD extraction yield of 40.12% and the diterpenoid content of 81.80%. In vivo studies revealed that TD attenuated renal injury, improved glucose and lipid dysmetabolism, and reduced renal oxidative stress in DKD mice. The in vivo absorption profiles showed that TD also exhibited good bioavailability.

**Conclusion:**

This study provides an effective method for TD enrichment and demonstrates the renoprotective effects of TD. These findings offer new insights on the broader use of rosemary leaves and the development of natural product-based therapies for DKD.

**Graphical Abstract:**

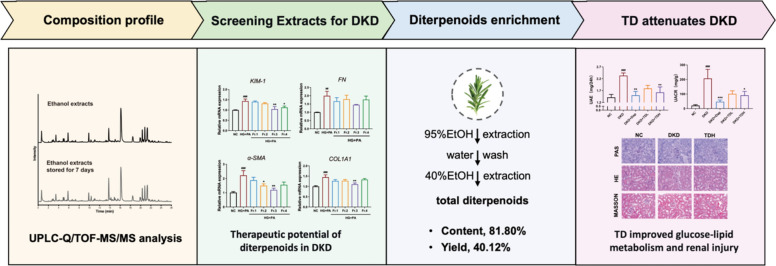

**Supplementary Information:**

The online version contains supplementary material available at 10.1186/s13020-026-01404-1.

## Introduction

*Rosmarinus officinalis* L. (rosemary) originally comes from the Mediterranean region. According to the *Compendium of Materia Medica,* rosemary has been classified as traditional Chinese medicine with pungent in flavor, warm in nature, and non-toxic. Traditionally, it has been used as a carminative, antispasmodic, analgesic, and promote hair growth. Current research suggested that rosemary possesses various potential therapeutic effects, including antibacterial, antidepressant, antitumor, anti-inflammatory, hypoglycemic and hypolipidemic effects [1–5].

The pharmacological activities of rosemary stem from the rich chemical components, including phenolic acids, diterpenoids, triterpenoids, and flavonoids [6, 7]. However, the key active components, particularly carnosic acid, are extremely sensitive to oxygen [8]. Conventional enrichment methods such as Soxhlet extraction and maceration, which are prone to cause the oxidative degradation of these components. Such inherent instability of rosemary leaves extracts limits its broader industrial applications. Therefore, improving the stability of these bioactive components during enrichment is a major challenge for the full application of rosemary.

To address these challenges, this study systematically investigated the pharmacodynamic material basis of rosemary leaves and established a novel enrichment method. Chemical profiles were characterized by ultra performance liquid chromatography quadrupole time-of-flight mass spectrometry (UPLC-Q/TOF–MS), and diterpenoids were identified as the principal active constituents responsible for the anti-DKD effects. Furthermore, study on degradation kinetics and elucidation of the degradation pathways of diterpenoids provided a basis for total diterpenoids (TD) enrichment method development. The new method used water and ethanol as solvents and vitamin C as a stabilizer. Importantly, vitamin C was completely removed in the final enrichment step. The optimized method effectively suppressed TD degradation and yielded an extract with a TD content above 80%.

In vivo studies showed that TD ameliorated glucose and lipid metabolism disorders and attenuated renal injury in DKD mice. TD also has been proven to alleviate renal oxidative stress and activate the transcription factor nuclear factor erythroid 2-related factor 2 (Nrf2)/heme oxygenase-1 (HO-1) signaling pathway. Pharmacokinetic studies further demonstrated that the major components of TD exhibit good bioavailability. This work represents a new strategy for production of rosemary diterpenoids thereby providing support for the further application of rosemary, as well as facilitating the development of potential drugs against DKD.

## Materials and methods

### Materials

Methanol, formic acid, and acetonitrile were purchased from Sigma-Aldrich (Shanghai, China). Vitamin C, ethanol, and phosphoric acid were obtained from Sinopharm Group Chemical Reagent (Shanghai, China). Milli-Q Water was prepared using a Milli-Q water purification system (USA).

Reference standards of carnosol, rosmanol, rosmarinic acid, homoplantaginin, cirsimaritin, and 12-metoxy-carnosic acid were purchased from Chengdu Must Biotechnology (Chengdu, China). Apigenin, hispidulin, epirosmanol, genkwanin, pectolinarigenin, bergenin, and betulinic acid were purchased from Chengdu Push Biotechnology (Chengdu, China). Carnosic acid was purchased from Chengdu Pufei De Biotech (Chengdu, China).

### Preparation of fraction 1–4

Rosemary leaves were purchased from Bozhou Traditional Chinese Medicine Market (Bozhou, China) and stored in the dark at 26 °C  ± 5 °C for 24 h. The collected samples were authenticated by Prof. Ping Li (China Pharmaceutical University). Dried rosemary leaves were subjected to two rounds of extraction using 95% ethanol at 90 °C. The resulting filtrate was concentrated and lyophilized, yielding a concentrated residue. Next, the crude extracts (Fraction-1) were partitioned sequentially by water (Fraction-2) and cyclohexane (Fraction-3). The residue was Fraction-4.

### Extraction of TD

A series of single-factor experiments was performed to evaluate the effects of various parameters on the extraction efficiency of TD. Extraction method, ethanol concentration, solid/liquid ratio, temperature, heat reflux time, and number of cycles were investigated. The content of diterpenoids in extracts was measured to select the optimal extraction conditions.

### Purification of TD

To optimize multiple factors and determine the optimal conditions with fewer experimental trials, we utilized an orthogonal experiment to optimize critical factors for the removal of phenolic acids. Single-factor experiments were conducted to screen the critical factors affecting the removal of phenolic acid. Subsequently, an L9 (3^3^) orthogonal experiment was employed to optimize critical factors, including washing method (A), solid/liquid ratio (B), and number of washing cycles (C) (Tables S1 and S2).

To remove triterpenoids, the extracts were diluted with ethanol solution and filtered to selectively isolate of triterpenoids. Ethanol concentration and solid/liquid ratio were evaluated. The yield and content of carnosic acid, and the area of betulinic acid were measured to determine the optimal conditions.

### Qualitative analysis of rosemary leaves extracts

The extracts and reference standards were diluted with methanol to a final concentration of 0.1 mg/mL. Following centrifugation at 13,000 rpm for 5 min, the resulting supernatant was stored at 4 °C as test solution.

Qualitative analysis was conducted using an Agilent 1290 Infinity UPLC system equipped with a 6530 Q-TOF system (Santa Clara, USA). UPLC method: The chromatographic separation was employed on an Agilent Zorbax Eclipse Plus C18 column (2.1 mm × 100 mm, 1.8 μm) using a gradient elution with solvent A (0.1% formic acid in water) and solvent B (acetonitrile) as follows: 0 min, 5%B; 2 min, 15%B; 13 min, 60%B; 18 min, 60%B; 19 min, 80%B; 27 min, 95%B; 27.5 min, 95%B; 28.5 min, 5%B; 33 min, 5%B. The flow rate was set at 0.4 mL/min, with an injection volume of 1 μL, and the column temperature was maintained at 30 °C. MS method: Mass spectrometric detection was conducted using a Dual AJS ESI source operated in negative ion mode under the following conditions: capillary voltage, 3500 V; fragmentor voltage, 120 V; nebulizer gas pressure, 35 psi; drying gas (N₂) temperature, 350 °C; drying gas flow rate, 10 L/min; sheath gas temperature, 350 °C . Collision energies of 20 V and 40 V were applied. The MS scan range was set to *m/z* 100–1500, and the auto MS/MS mode covered *m/z* 100–1000.

### Quantitative analysis of TD

10.00 mg of extracts were dissolved in 25 mL methanol. The solution was centrifuged at 13,000 rpm for 5 min, the resulting supernatant was stored at 4 °C for subsequent analysis. Reference standards were prepared at a concentration of 1 mg/mL in methanol and further diluted to appropriate concentration ranges (Table S4).

Quantitative analysis was performed using an Agilent 1290 Infinity UPLC system equipped with a diode array detector (DAD). The UPLC method was consistent with description in Section "Qualitative analysis of rosemary leaves extracts". The injection volume was 2 μL, and detection was carried out at a wavelength of 284 nm. Quantification was determined by the standard curve method using Microsoft Excel.

### Extraction yield

The extraction yield of TD was evaluated by the content of carnosic acid and carnosol. The formula is as follows:

$${\text{X (\% ) = M}} \times {\mathrm{C}}\,{\mathrm{/}}\,{\mathrm{(M0}}\,\, \times \,\,C0)\,\, \times \,\,100$$

where *X* represents the extraction yield, *M* (g) is the mass of the extracted TD, *C* (%) is the content of carnosic acid and carnosol in extracted TD. *M*_*0*_ (g) is the dried rosemary mass, *C*_*0*_ (%) is the content of carnosic acid and carnosol in rosemary leaves.

### Quantitative degradation kinetics

The detailed methods for the thermal degradation kinetics of carnosic acid under different heating conditions are provided in the Supplementary Material.

### Stability improvement of TD

The effects of phosphoric acid, formic acid, and vitamin C on the stability of carnosic acid in the TD solution were investigated. The concentration of vitamin C was further optimized. After selecting vitamin C as the stabilizer, the stability of the TD solution was evaluated over one month at 4 °C, 25 °C, and 50 °C, which was assessed by the content of carnosic acid. To investigate potential chemical interactions between vitamin C and diterpenoids, UPLC-Q/TOF–MS analysis was performed on carnosic acid and TD with or without vitamin C after 30 days of storage.

### Cell culture

The HK2 cells were incubated in the MEM medium containing 10% FBS, 100 μg/mL streptomycin, and 100 μg/mL penicillin at 37 °C with 5% CO_2_. The cells were stimulated with high glucose (30 mM) and palmitic acid (200 μM) with or without combination of extracts (8 μg/mL) for 24 h.

### Cell viability assay

To evaluate the effect of extracts on the viability of HK2 cells, cells were seeded in 96-well plates (1 × 10^4^ cells) and incubated for 24 h. Subsequently, cells were treated with different concentrations of extracts. After an additional 24 h incubation, the media was discarded, and then 100 μL (90 μL cell media + 10 μL CCK8) of CCK8 mix solution (Abbkine, Wuhan, China) was added to the 96-well plate. Cells were then incubated for an additional 0.5 h. The cell viability was determined by measuring absorbance at 450 nm.

### Triacylglycerol and cholesterol measurement

To measure intracellular total triacylglycerol (TG) and total cholesterol (TC), cells were cultured in 6-well plates and harvested in 1 mL of PBS. A 100 µL aliquot of cell suspension was transferred into a new tube and centrifuged at 1000 × g for 5 min at 4 °C. Subsequently, this cell pellet was lysed in lysis buffer (Beyotime, Shanghai, China) and used for protein quantification (BCA protein assay kit, Jiancheng, Nanjing, China). The remaining cell suspension was used for lipid extraction. After centrifugation at 1000 × g for 5 min at 4 °C, the collected cell pellet was thoroughly mixed with 200 μL of chloroform/methanol (2:1, v/v) on a shaker for 3 h at 24 °C . 100 μL NaCl solution (0.1 M) was added to each reaction tube and mixed thoroughly, followed by centrifugation at 3700 rpm for 10 min. The lower organic phase was collected and evaporated to dryness. The dried residue was resuspended in 10 μL of 1% Triton X-100 in anhydrous ethanol, and the concentrations of TG and TC were determined using commercial assay kits (Jiancheng, Nanjing, China) according to the manufacturer’s instructions.

### RT–qPCR

Total RNA was isolated from sample using Trizol reagents (Vazyme, China) and reverse transcribed into cDNA using HiScript Reverse Transcriptase kit (Vazyme, China). Quantitative real-time PCR using SYBR Green Master Mix (Vazyme, China) was performed with Light-Cycler 480 (Roche Diagnostics GmbH). The primer sequences used in this study are listed in Table S7.

### Animal experiment

All animal experiments were approved by the Institutional Animal Care and Use Committee (IACUC) of China Pharmaceutical University (No.202409032) and were conducted in accordance with the National Institutes of Health Guide for the Care and Use of Laboratory Animals (No. 8023). Animals were housed under standard conditions (22 ± 2 °C, 12-h light/dark cycle) with ad libitum access to food and water. Humane endpoints were strictly applied based on body weight loss and mouse daily activity. Animals were randomly assigned to experimental groups using a random number generator to minimize selection bias. The drug administration was not blinded to group allocation due to the nature of the intervention. However, the outcome assessment and data analysis were performed by investigators blinded to group allocation.

#### In vivo therapeutic effects of TD on DKD mice model

The C57BL/6J mice were randomly divided into the standard chow diet (Jiangsu synergetic biology, 1,010,039) group and the high fat diet (HFD, 60% fat, Research Diets, D12492) group. After exposure for 4 weeks to the respective diets, the HFD-fed mice were injected intraperitoneally with 40 mg/kg streptozocin (STZ, Sigma-Aldrich) solution after overnight fasting for 5 consecutive days. The chow group received the vehicle only. After 7 days, the HFD/STZ mice with fasting blood glucose levels ≥ 16.7 mM were induced successfully. Mice were randomly divided into 5 groups, with 10 mice in each group, as follows: (i) NC group (normal mice treated with 0.5% CMC-Na); (ii) DKD group (DKD mice treated with 0.5% CMC-Na); (iii) DKD + Dap group (DKD mice treated with dapagliflozin); (iv) DKD + TDL group (DKD mice treated with 25 mg/kg TD); and (v) DKD + TDH group (DKD mice treated with 50 mg/kg TD). The treatments were administered once daily for 10 weeks. The dosages of TD were chosen based on previous studies [4]. The dosage of dapagliflozin was calculated by body surface area normalization method (Meeh-Rubner formula).

At the end of the intervention, level of fasting blood glucose was measured by glucometer (Sinocare, China). 24-h urine was collected and centrifuged, and the supernatant was analyzed for urinary albumin, urine microalbumin, and urinary creatinine by using the corresponding assay kit (Jiancheng, Nanjing, China). Serum samples were extracted to detect the levels of creatinine, blood urea nitrogen (BUN), TG, TC, low density lipoprotein cholesterol (LDL-C), and high-density lipoprotein cholesterol (HDL-C) using the corresponding assay kits (Jiancheng, Nanjing, China). Malondialdehyde (MDA), superoxide dismutase (SOD), and glutathione peroxidase (GSH-Px) were quantified utilizing assay kit from Bioswamp (Wuhan, China).

#### The in vivo absorption profile of the TD

The experimental procedures, including animal group, administration, preparation of plasma samples, analysis of plasma samples and analytical method validation, are described in detail in the Supplementary Material.

#### Acute toxicity of TD

Balb/c mouse (8 weeks, half male and half female) were purchased from Charles River Laboratory (Pinghu, China). Mice were randomly divided into 6 groups (n = 10 per group; 5 males and 5 females). Details of mice grouping and administration doses are presented in Table. S14. Mice received a single oral administration of TD and general observations for 14 days. On day 14, all surviving animals were euthanized, and major organs (heart, liver, spleen, lungs, kidneys, and small intestine) were collected for histological analysis using hematoxylin and eosin (H&E) staining.

### Insulin tolerance and oral glucose tolerance tests

At the end of the intervention, insulin tolerance test (ITT) was performed. The level of blood glucose was measured by venous bleeding at 0, 30, 60, 90, and 120 min after *i.p.* injection of insulin at 0.75 IU/kg. After 3 days, oral glucose tolerance test (OGTT) was investigated. The blood glucose levels were measured at 0, 15, 30, 60, 90 and 120 min after oral glucose of 2 g/kg. Area under the curve (AUC) was calculated to quantify the ITT and OGTT results.

### Histopathological examination

The kidneys were removed at the end of the intervention and rinsed in pre-cooled saline. Half of the kidneys were fixed in a 4% paraformaldehyde solution for 48 h and embedded in paraffin. Tissue sections were prepared and subjected to pathological evaluation using H&E, periodic acid–Schiff (PAS), and Masson trichrome staining. Images were captured with an OLYMPUS IX73 microscope (Tokyo, Japan).

### Western blot

Mice kidney tissues were lysed on ice in RIPA (Beyotime, Shanghai, China) buffer with protease inhibitors (Roche, Shanghai, China), followed by 10 min of centrifugation at 12,000 g at 4 °C to obtain the supernatant. Protein concentration was determined by a protein quantification (BCA protein assay kit, Jiancheng, Nanjing, China). Protein (20 μg/lane) was electrophoresed on 10% SDS polyacrylamide gel (Servicebio, Wuhan, China) and transferred to a 0.22 μm NC membrane (Millipore, USA), then blocked with 5% bovine serum albumin for 1 h. After washing with TBST thrice, all membranes were incubated overnight with the following primary antibodies: Nrf2 (1:1000, Cell signaling Technology, 12,721), HO-1 (1:5000, proteintech, 10,701–1-AP), and β-actin (1:1000, proteintech, 66,009–1-Ig). They were then incubated with secondary antibodies at ambient temperature for 1 h. Quantification was performed using the ECL kit (Abbkine, Wuhan, China).

### Statistical analysis

All data are expressed as means ± SEM. The comparisons between groups were made using one-way ANOVA (Dunnett’s posttest). At least 3 independent experiments were performed. Statistical analysis was performed by GraphPad Prism 10.3.1. Differences with *P* < 0.05 were considered as statistically significant.

## Results

### Chemical characterization of rosemary leaves extracts.

**Fig. 1:**
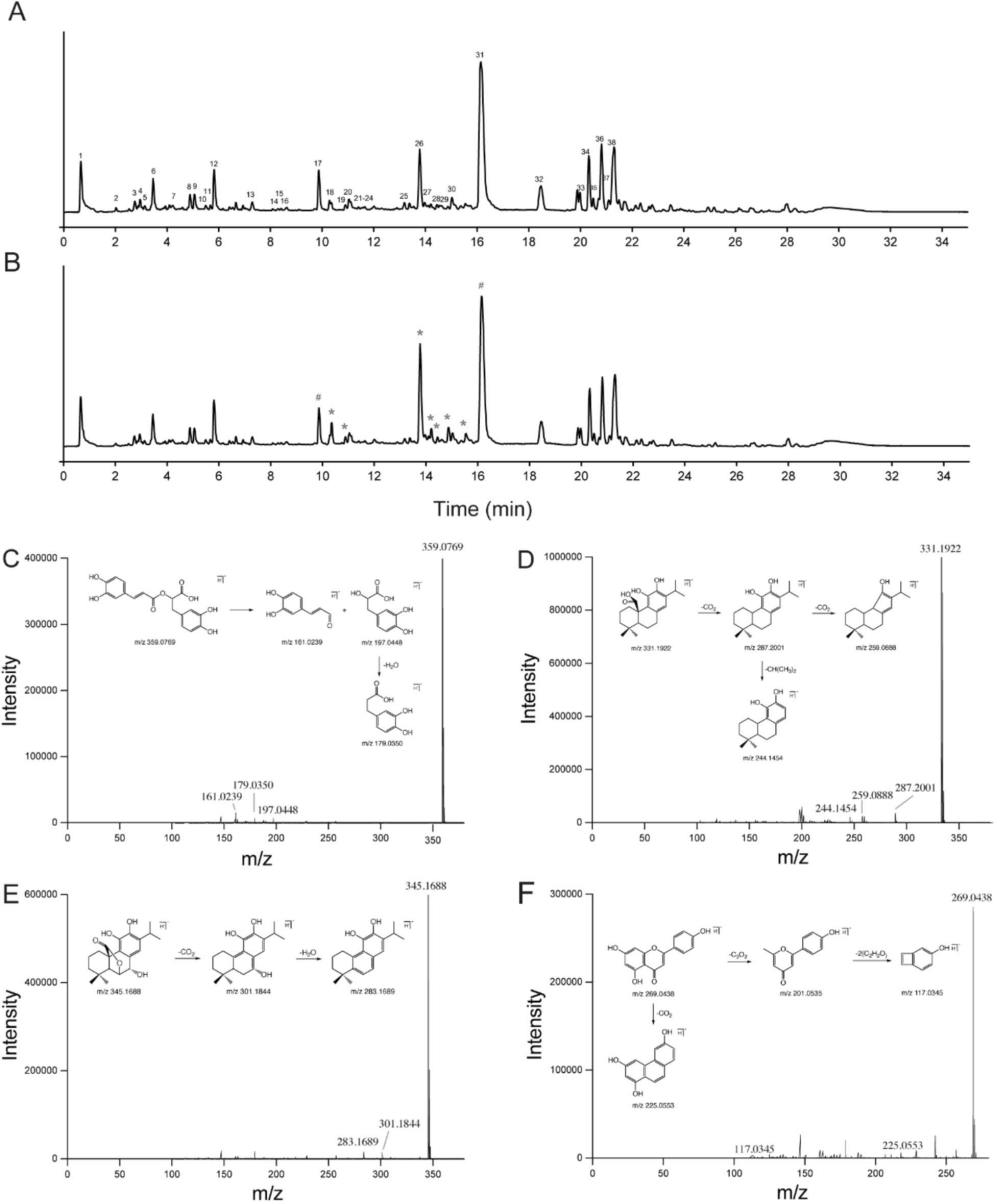
UPLC-Q/TOF–MS/MS analysis of rosemary leaves extract. **A**, **B** Total ion chromatograms (TIC) in negative mode. (**A)** Rosemary leaves 95% ethanol extracts; the numbers corresponding compounds are shown in Table 1. (**B)** Rosemary leaves 95% ethanol extracts after 7 days of storage at room temperature. ^#^ denotes degradable compounds, and ^*^ denotes degradation products shown in Table S8. **C**–**F** Proposed fragmentation pathway for (**C)** rosmarinic acid; (**D)** carnosic acid; (**E)** epirosmanol; (**F)** apigenin

The chemical composition of 95% ethanol extracts of rosemary leaves was analyzed by UPLC-Q/TOF–MS/MS, resulting in the identification of 38 compounds based on the retention time, molecular weights, fragment ions, and comparison with reference standards. The typical total ion chromatogram (TIC) in negative mode is shown in Fig. 1A. As summarized in Table 1, the extracts were primarily composed of phenolic acids, flavonoids, diterpenoids, and triterpenoids.

**Table 1:** Peak assignments of rosemary leaves extracts

Peak	t_R_ (min)	Identification	[M-H]^−^ (m/z)	Molecular formula	MS/MS^2^ fragment ions	Types
1	0.665	Quinic acid	215.0384	C_7_H_12_O_6_	127.0398	Others
2	2.064	Syringic acid	197.0441	C_9_H_10_O_5_	167.0339	Phenolic acid
3	2.731	Caffeic acid 3-glucoside	341.086	C_15_H_18_O_9_	281.0643、179.0341、161.0231	Phenolic acid
4	2.984	Caffeic acid 3-glucoside isomer	341.0847	C_15_H_18_O_9_	281.0643、179.0341、161.0231	Phenolic acid
5	3.474	Gallocatechin	305.0685	C_12_H_18_O_4_	146.9656	Flavonoid
6	3.584	Caffeic acid	179.0341	C_12_H_18_O_4_	135.0448	Phenolic acid
7	4.234	Quercetin 3-β-D-glucoside	463.0845	C_21_H_20_O_12_	301.0383	Flavonoid
8	4.947	Rosmarinic acid 3-O-glucoside	521.1279	C_24_H_26_O_13_	161.0031	Phenolic acid
9	5.047	Nepetrin	477.0983	C_22_H_22_O_12_	315.0481、299.0170	Flavonoid
10	5.497	Apigenin-7-O-glucoside	431.0945	C_21_H_20_O_10_	269.0439	Flavonoid
11^*^	5.664	Homoplantaginin	461.1057	C_22_H_22_O_11_	299.0532、283.0228	Flavonoid
12^*^	5.88	Rosmarinic acid	359.0769	C_18_H_16_O_8_	197.0448, 179.0336, 161.0239	Phenolic acid
13	7.28	Isorhamnetin	315.0482	C_16_H_12_O_7_	300.0255	Flavonoid
14^*^	8.28	Apigenin	269.0438	C_15_H_10_O_5_	225.0553、151.0025、117.0345	Flavonoid
15^*^	8.413	Hispidulin	299.0544	C_16_H_12_O_6_	284.0313、136.9873	Flavonoid
16^*^	9.863	Cirsimaritin	313.0707	C_17_H_14_O_6_	298.0494、283.0260	Flavonoid
17^*^	9.863	Epirosmanol	345.1688	C_20_H_26_O_5_	301.1844、283.1689、267.1414	Diterpenoid
18^*^	10.363	Rosmanol	345.1689	C_20_H_26_O_5_	283.1711、227.1084	Diterpenoid
19	10.912	Epiisorosmanol	345.1686	C_20_H_26_O_5_	283.1677	Diterpenoid
20^*^	11.046	Genkwanin	283.0597	C_16_H_12_O_5_	268.0355	Flavonoid
21^*^	11.228	Pectolinarigenin	313.0695	C_17_H_14_O_6_	298.0461、283.0235、163.0026	Flavonoid
22^*^	11.412	Acacetin	283.0596	C_16_H_12_O_5_	268.0361	Flavonoid
23	11.529	Rosmadial	343.1535	C_20_H_24_O_5_	315.1589、299.1634、287.1640	Diterpenoid
24	11.646	Rosmanol isomer	345.1693	C_20_H_26_O_5_	301.1791	Diterpenoid
25	13.178	Columbaridione	343.1529	C_20_H_24_O_5_	299.1631、271.1719	Diterpenoid
26^*^	13.778	Carnosol	329.1739	C_20_H_26_O_4_	285.1848	Diterpenoid
27	14.212	Carnosic acid quinone	329.1729	C_20_H_26_O_4_	285.1846	Diterpenoid
28	14.545	Carnosol quinone	343.1527	C_20_H_24_O_5_	315.1589、299.1634	Diterpenoid
29	14.878	Epirosmanolethylether	373.198	C_22_H_30_H_5_	329.1725、283.1678、227.1059	Diterpenoid
30	15.045	Rosmaridiphenol	315.1928	C_20_H_28_O_3_	285.1837	Diterpenoid
31^*^	16.178	Carnosic acid	331.1922	C_20_H_28_O_4_	287.2001、244.1454	Diterpenoid
32^*^	18.482	12-metoxy-carnosic acid	345.2044	C_21_H_30_O_4_	301.2184、286.1947、271.1708	Diterpenoid
33	19.977	9-shogaol	317.2099	C_20_H_30_O_3_	287.2002	Others
34	20.327	Micromeric acid	[M + H]^+^ 455.3640	C_30_H_46_O_3_	[M + H]^+^ 409.3571	Triterpenoid
35	20.493	1-phenanthrenecarboxylic acid	299.2003	C_20_H_28_O_2_	283.1685、243.1381	Others
36	20.81	Betulinic acid	455.3504	C_30_H_48_O_3_	−	Triterpenoid
37	21.08	Oleanolic acid	455.3493	C_30_H_48_O_3_	−	Triterpenoid
38^*^	21.31	Ursolic acid	455.3488	C_30_H_48_O_3_	−	Triterpenoid

^*^Identification of the compound was confirmed by

### Identification of phenolic acids

Compounds 2, 3, 4, 6, 8, and 12 were identified as syringic acid, caffeic acid 3-glucoside, caffeic acid 3-glucoside isomer, caffeic acid, rosmarinic acid 3-O-glucoside, and rosmarinic acid, respectively. The proposed fragmentation pathway of rosmarinic acid is shown in Fig. 1C**.** Rosmarinic acid produced the [M-H]^−^ ion at *m/z* 359.0769. This result indicated a fragmentation pattern corresponding to the ester bond cleavage, yielding characteristic fragmented ions at *m/z* 179.0350 and 197.0448. The ion at *m/z* 161.0239 was produced by the loss of a H_2_O molecule from the *m/z* 197.0448 fragment.

### Identification of diterpenoids

Thirteen diterpenoids were characterized in rosemary leaves in this study. Compound 26 ([M-H]^−^ at *m/z* 329.1739) and compound 31 ([M-H]^−^ at *m/z* 331.1922) were identified as carnosol and carnosic acid, respectively. Carnosic acid produced the major fragment ion at *m/z* 287.2001 corresponding to the loss of CO_2_ fragment. The fragment ion at *m/z* 244.1454 resulted from the further loss of CH(CH_3_)_2_ from [M-H-CO_2_]^−^ ion. Similar fragmentation patterns were observed for carnosol, which produced a major fragment ion at *m/z* 285.1848 due to the loss of CO_2_. Epirosmanol, rosmanol, and epiisorosmanol are isomers which produce [M-H]^−^ ions at *m/z* 345 and share the same fragmentation pattern characterized by two prominent ions at *m/z* 301 and *m/z* 283. They were further confirmed by comparison of the retention time with reference standards. The proposed fragmentation pathways of carnosic acid and epirosmanol are shown in Fig. 1D and E, respectively.

### Identification of flavonoids

Twelve flavonoids were characterized in rosemary leaves, most of which contain a core 2-phenyl-5-hydroxychromone skeleton, exemplified by homoplantaginin and apigenin. Homoplantaginin produced a primary [M-H]^−^ ion at *m/z* 461.1057, with fragment ions observed at *m/z* 299.0532 and 283.0228, corresponding to [M–H-C_6_H_11_O_5_]^−^ and [M-H-C_6_H_11_O_5_-OH]^−^ fragments, respectively. Compound 14 was identified as apigenin by comparison with the reference standard. The precursor ion ([M-H]^−^ at *m/z* 269.0438) lost the CO_2_ to produce a fragment ion at *m/z* 225.0553. Further fragmentation yielded an ion at *m/z* 117.0345 through continuous loss of C_3_H_2_ and two C_2_H_2_O units (Fig. 1F).

### Diterpenoids alleviated lipid accumulation and fibrosis in HG/PA-stressed HK2 cells

**Fig. 2:**
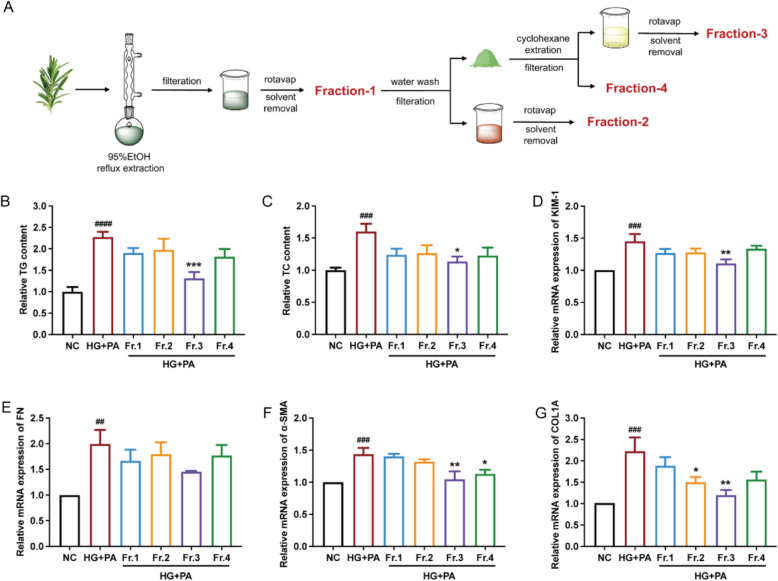
Screening of major anti-DKD constituents. **A** Enrichment workflow of different fractions from rosemary leaves. **B**–**G** HK2 cells were stimulated with HG and PA for 24 h with or without treatment with different fractions. The levels of total TG (**B**), TC (**C**) content; The mRNA expression levels of KIM-1 (**D**), FN (**E**), α-SMA (**F**) and COL1A1 (**G**) were determined by RT–qPCR. (n = 3). ^*^P < 0.05, ^**/##^*P* < 0.01, ^***/###^*P* < 0.001, ^****/####^*P* < 0.0001. Data are shown as Mean ± SEM. Statistical analysis was performed by one-way ANOVA, ^#^denotes comparison with NC group, and ^*^ denotes comparison with model group

Rosemary has been reported to exhibit therapeutic potential against DKD. To identify its active constituents, four fractions (Fr.1-Fr.4) were obtained by solvent extraction and subsequently characterized using UPLC-Q/TOF-MS/MS (Fig. 2A). The results showed that Fr.1 was the crude extract, while Fr.2 and Fr.3 were predominantly phenolic acids and diterpenoids, respectively. Fr.4 primarily contained both triterpenoids and diterpenoids (Fig. S1A). The effects of various extracts on cell viability are shown in Fig. S1B–E.

Lipid accumulation in renal tubular epithelial cells represents a hallmark of DKD [9]. To assess the effects of rosemary fractions on lipid accumulation, we assessed TG and TC levels in HK2 cells exposed to high glucose (HG) and palmitic acid (PA). The results showed that all fractions attenuated lipid accumulation, with Fr.3 exhibiting the most significant effect in reducing TG and TC levels (Fig. 2B, C).

To investigate whether the extract fractions could reverse renal injury and renal fibrosis, the key biomarkers were evaluated. As shown in Fig. 2D, KIM-1, a biomarker of renal injury, was significantly down-regulated following treatment with Fr.3. Moreover, the expression of key fibrosis-related genes (FN, α-SMA, and COL1A1) was downregulated in HK2 cells following treatment with Fr.3 (Fig. 2E-G), suggesting that diterpenoids are the primary active components against DKD.

### Enrichment of diterpenoid fraction

**Fig. 3:**
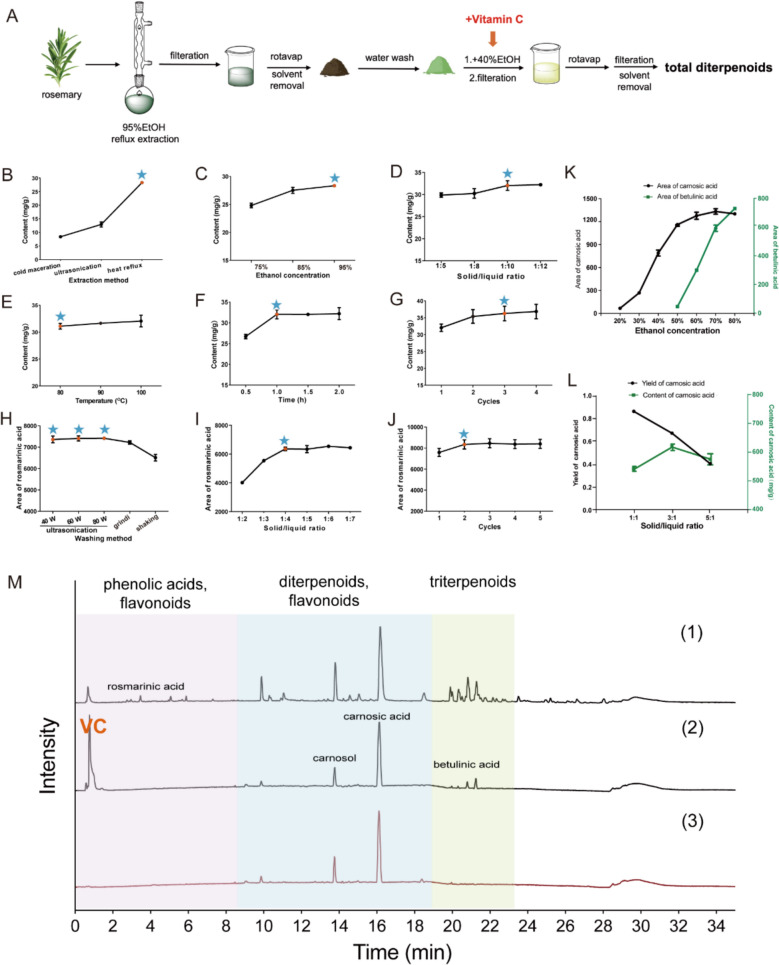
Enrichment of TD. **A** Enrichment workflow of TD. **B**–**G** Effect of the different variables on the extraction of TD. (**B)** extraction method; (**C)** ethanol concentration; (**D)** solid/liquid ratio; (**E)** heating temperature; (**F)** heating reflux time. (**G)** the number of cycles. **H–L** Effect of the different variables on the purification efficiency of TD. For the removal of phenolic acids, (**H)** washing method; (**I)** solid/liquid ratio; (**J)** the number of cycles. For the removal of triterpenoids, **K** ethanol concentration; **L** solid/liquid ratio. **M** Total ion chromatograms in negative mode of: (1) Rosemary leaves 95% ethanol extracts; (2) The extracts after water washing and addition of vitamin C; (3) Final products of TD. Data are shown as Mean ± SEM. Statistical analysis was performed using one-way ANOVA

The rosemary diterpenoids were extracted with 95% ethanol, followed by aqueous washing and purification with 40% ethanol. The enrichment process is shown in Fig. 3A**.** Corresponding chromatograms are shown in Fig. 3M.

#### Extraction

A single-factor experiment was conducted to obtain the maximal diterpenoid yield. The content of carnosol and carnosic acid, which are representative diterpenoids in rosemary leaves, were used as criteria to evaluate extraction efficiency. Comparation of different variables in single-factor experiment of extraction optimization is shown in Table S5.

A comparative evaluation of extraction methods revealed that heat reflux exhibited superior efficiency compared to cold maceration and ultrasonication (Fig. 3B). This extraction performance likely comes from the combined effects of heating and solvent recycling. The elevated temperature accelerates molecular motion and promotes the diffusion of target compounds into the solvent. Simultaneously, the continuous solvent recycling facilitates repeated interaction between the target compounds and solvent. Heat reflux was selected as the preferred method for subsequent optimizations.

Ethanol concentration exhibited a positive relationship with extraction efficiency. This is likely due to the lipophilic nature of diterpenoids. Higher ethanol concentration improves their solubility and thus increases extraction yield. Herein, 95% (v/v) ethanol was selected as the optimal solvent for further study (Fig. 3C).

Due to the concentration gradient between the solvent and target compounds, extraction efficiency also rose with increasing solid/liquid ratio until mass transfer equilibrium was reached. As shown in Fig. 3D, the extraction efficiency of diterpenoids increased by higher solid/liquid ratios and reached saturation of 33.4 mg/g at 1:10 (g/mL). Further increase in solid/liquid ratios did not result in higher content. Accordingly, 1:10 (g/mL) was selected as the optimal ratio.

Under extraction temperature of 80 °C, 90 °C, and 100 °C, no significant difference in extraction efficiency was observed. Thus, 80 °C was selected for the extraction (Fig. 3E), Heat reflux time and the number of cycles affected extraction efficiency, with yield reaching maximum after 1 h of heat reflux and 3 extraction cycles (Fig. 3F and G).

In summary, these results suggested that the optimal extraction conditions for TD were as follows: extraction method, heat reflux; solvent, 95% (v/v) ethanol; solid/liquid ratio, 1:10 (g/mL); temperature, 80 ℃; heat reflux time, 1 h; and number of extraction cycles, 3.

#### Purification

As summarized in Table 1, rosemary leaves extracts contain phenolic acids, flavonoids, diterpenoids, and triterpenoids. The elution order of these compounds on the C18 column suggested a progressive decrease in polarity [10].

1. Removal of phenolic acids

The favorable water solubility of phenolic acids enabled their selective isolation through water washing. As the representative phenolic acid, the peak area of rosmarinic acid in the washing solution was determined as the criterion for the optimal conditions.

The washing method was considered as a key step influencing removal efficiency. As shown in Fig. 3H, ultrasonication resulted in higher phenolic acid removal efficiency compared to both grinding and shaking. And variation of ultrasonic frequency had only a negligible effect on removal efficiency. Moreover, solid/liquid ratio also influenced the removal efficiency of phenolic acids. As shown in Fig. 3I, rosmarinic acid content initially increased and reached saturation at a ratio of 1:4 (w/v). A similar trend was observed with the number of washing cycles, where the rosmarinic acid content increased within a limited range before reaching a plateau (Fig. 3J).

Subsequently, an orthogonal experiment was employed based on single-factor experiment, focusing on three factors: washing method (A), solid/liquid ratio (B), and number of washing cycles (C), with each factor set at three levels. According to the variance analysis results presented in Table S3, the washing method showed statistically significant effects on the phenolic acid removal efficacy. The range analysis (r) in Table S2 suggests that the factors involving phenolic acids removal efficiency follow the order A > C > B. Accordingly, the optimal factor combination was determined as “A1B2C2”, corresponding to ultrasonication, a solid/liquid ratio of 1:3, and three wash cycles.

(2) Removal of triterpenoids

Triterpenoids present lower polarity compared to diterpenoids, they could be selectively isolated by ethanol extraction and filtration. In Fig. 3K, the peak areas of carnosic acid and betulinic acid were measured as representative components for diterpenoids and triterpenoids, respectively. The extraction efficiency of diterpenoids rose steadily as the ethanol concentration increased from 20 to 50%. By contrast, the extraction efficiency of triterpenoids increased sharply once the ethanol concentration exceeded 40%. Therefore, 40% ethanol was selected as the optimal solvent to remove triterpenoids.

The optimal solid/liquid ratio was determined by evaluating yield and content of diterpenoids in the final product. A ratio of 3:1 (g/mL) was identified as a suitable condition for triterpenoids removal (Fig. 3L). Consequently, 40% ethanol was added to the extract at a ratio of 3:1 (g/mL). Based on this, diterpenoids were selectively extracted into the 40% ethanol phase, while triterpenoids remained in the residue and were isolated by filtration. Ethanol was removed by rotary evaporation. The concentrated solution was filtered again, and the resulting residue was vacuum-dried to obtain the TD extracts.

### Degradation profiles of rosemary leaves extracts.

**Fig. 4:**
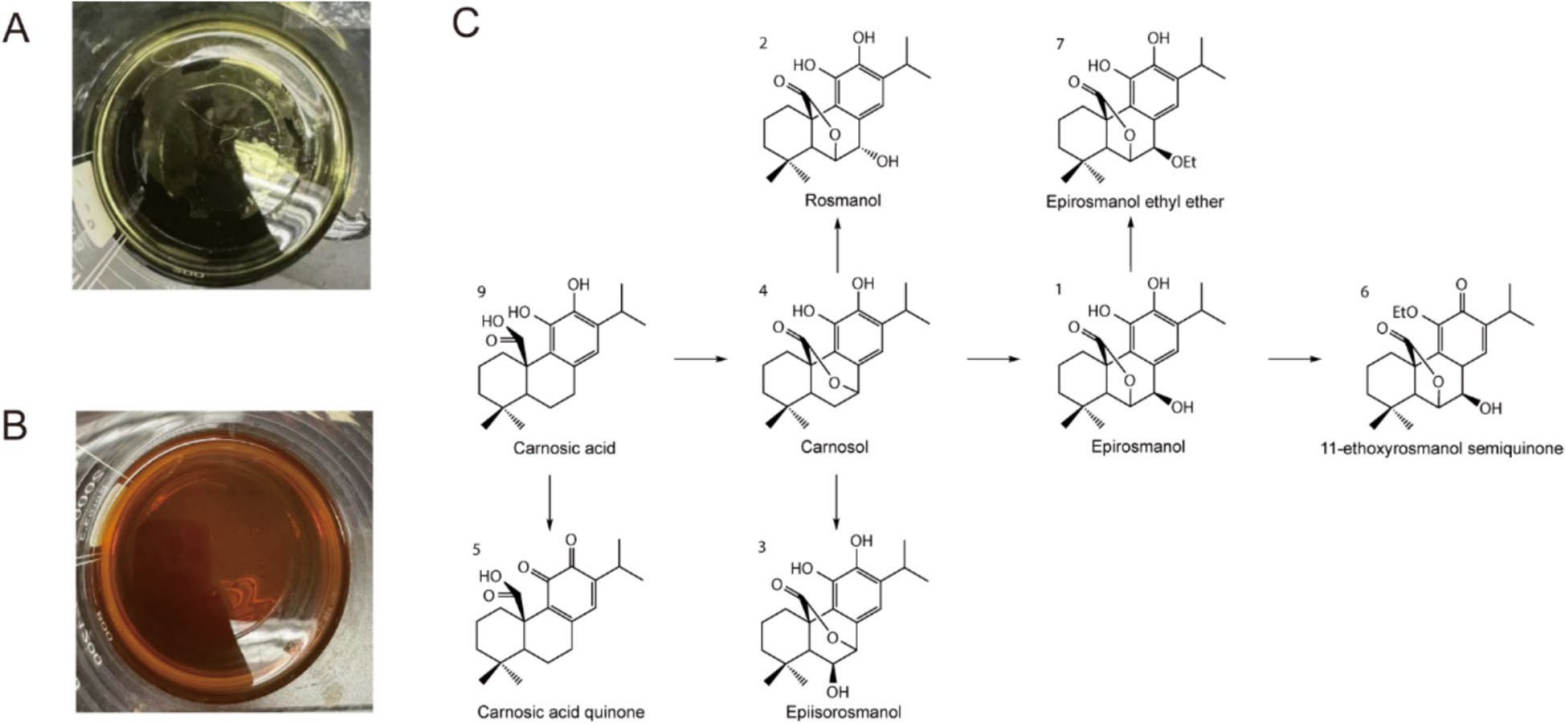
Degradation of Rosemary leaves extracts. **A** Photograph of rosemary leaves extracts. **B** Photograph of rosemary leaves extracts after 5 h of storage at room temperature. **C** The proposed degradation process of diterpenoids

During the enrichment process, the nature of rosemary leaves ethanol extracts changed noticeably from green to orange (Fig. 4A and B). This color shift was attributed to the degradation of main components.

To investigate the degradation products, the extracts were analyzed using UPLC-Q/TOF–MS/MS after storage at room temperature for 7 days. As shown in Fig. 1B, chemical characterization revealed significant degradation of diterpenoids, particularly carnosic acid, which is known as a potent antioxidant [11]. The phenolic hydroxyl groups at C11 and C12 positions of carnosic acid enable it to act as a hydrogen donor, reacting with free radicals and terminating radical chain reactions [12]. However, the property also makes carnosic acid prone to oxidative degradation. As shown in Fig. 1B, the reduction in carnosic acid content was accompanied by a corresponding increase in carnosol content, suggesting a conversion between the two compounds [13]. The increased levels of rosmanol and epiisorosmanol suggested that they were likely derived from carnosol, which is a secondary product formed through the oxidation of carnosic acid [8, 14]. Two compounds eluting at 14.467 min and 14.906 min were characterized as 11-ethoxyrosmanol semiquinone and epirosmanol ethyl ether, respectively. They are putative derivatives of epirosmanol. The degradation compounds identified in the rosemary leaves extracts are summarized in Table S8. And the proposed degradation pathway is depicted in Fig. 4C.

### Stability improvement

During triterpenoid isolation, ethanol was removed by rotary evaporation with heating. This prolonged thermal exposure accelerates oxidation of carnosic acid. The thermal degradation kinetics of carnosic acid were systematically investigated. Firstly, the degradation of carnosic acid was assessed under different heating conditions. As shown in Fig. S2A, carnosic acid remained relatively stable at 4 °C and its degradation rate increased with increasing heat temperature. The degradation kinetic results suggest that carnosic acid degradation follows zero-order kinetics (Table S6). The corresponding degradation curves at different temperatures are shown in Fig. S2B, indicating that the thermal degradation rate of carnosic acid kept a constant rate at different concentrations.

Acidification of the extracts was initially used to reduce the degradation of carnosic acid. As shown in Fig. S2C, phosphoric acid slightly delayed the degradation process. In contrast, vitamin C (1 M) can effectively halt the oxidation of carnosic acid for up to 7 days (Fig. S2D). Importantly, vitamin C was readily removed by filtration in the last enrichment step due to its water solubility.

The stability of TD containing vitamin C was further evaluated over one month at 4 °C, 25 °C, and 50 °C. The results showed that it remained stable for up to 1 month at 4 °C and 25 °C, whereas carnosic acid showed degradation after 18 days at 50 °C (Fig. S2E).

To investigate potential chemical interactions between vitamin C and diterpenoids, UPLC-Q/TOF–MS analysis was performed on carnosic acid and TD with or without vitamin C after 30day storage. As shown in Fig. S2F, no new peaks were observed in the samples containing vitamin C, indicating that no new compounds were generated upon exposure to vitamin C.

### Identification and quantification of TD

The chemical composition of TD was characterized by UPLC-Q/TOF–MS/MS. A total of ten components with relatively high content were quantified by standard curve method, five of which were diterpenoids, making up 81.80% of TD. The predominant diterpenoids were identified as carnosic acid (59.11%), epirosmanol (12.60%), carnosol (6.43%), 12-metoxy-carnosic acid (3.17%), and rosmanol (0.49%), with the percentages representing their relative abundances in the extract (Table S4). The extraction yield was 41.12% under optimal conditions. The quantification analysis further demonstrated the reliability and efficiency of the enrichment method.

#### TD attenuates kidney injury and protects renal function

**Fig. 5:**
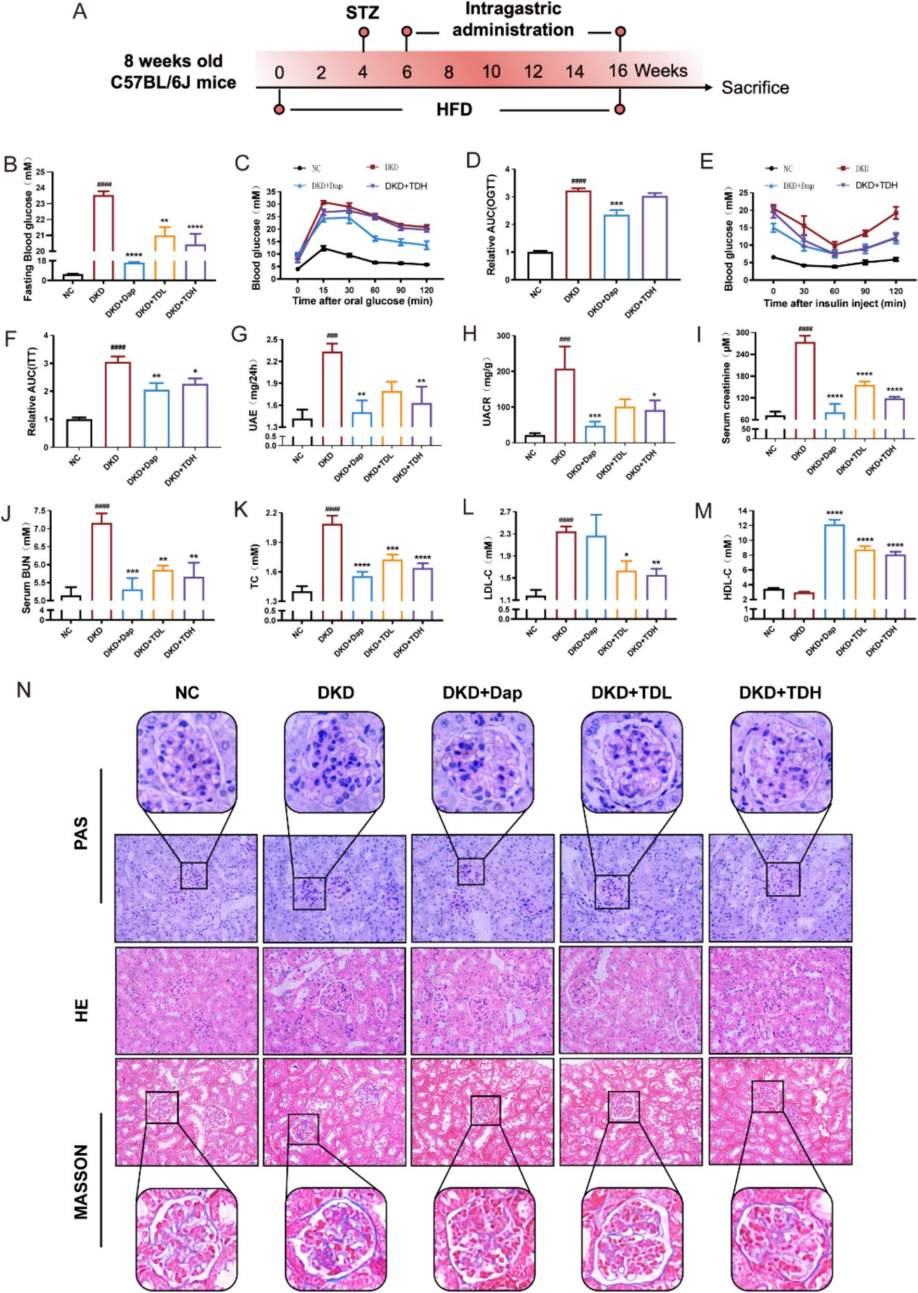
TD administration alleviates renal pathological damage and protects renal function in DKD mice. **A** Schematic overview of the experimental design of the mice model. **B** Fasting blood glucose levels of mice at the end of the intervention (*n* = 10). **C–F** Results of the OGTT and the ITT of mice in the indicated group after treatment with TD (*n* = 5). **G–N** Blood and urine samples were collected to detect UAE (**G**), UACR (**H**), serum creatinine (**I**), serum BUN (**J**), TC (**K**), LDL-C (**L**), HDL-C (**M**) at the end of the intervention (*n* = 10). **N** Kidney tissues were prepared into paraffin sections and subjected to PAS, H&E, and Masson trichrome staining (*n* = 6). Data were shown as Mean ± SEM. Statistical analysis was performed using one-way ANOVA, ^*^P < 0.05, ^**/##^*P* < 0.01, ^***/###^*P* < 0.001, ^****/####^*P* < 0.0001. “^#^” denotes comparison with NC mice, and “^*^” denotes comparison with DKD mice

We next evaluated the renoprotective effects of TD by DKD mouse model. A schematic overview of the experimental design is shown in Fig. 5A. As shown in Fig. 5B–F, TD treatment improved fasting blood glucose and insulin tolerance but had no effect on oral glucose tolerance in DKD mice. These findings suggest that TD can improve insulin sensitivity.

TD treatment also improved key markers of renal injury. 24-h urinary albumin excretion (UAE) and urine microalbumin-to-creatinine ratio (UACR) were markedly ameliorated by TD treatment (Fig. 5G and H). These improvements indicate that TD alleviates glomerular barrier dysfunction, a central event in early DKD progression. TD administration also reduced serum creatinine and BUN levels (Fig. 5I and J), indicating effective restoration of renal filtration function in DKD mice.

Lipid dysmetabolism is a well-recognized characteristic of DKD. As shown in Fig. 5K–M, TD reduced TC and LDL-C levels, accompanied by an increase in HDL-C, indicating that TD alleviates dyslipidemia in DKD mice.

To investigate the renal pathological changes following TD administration, H&E, PAS, and Masson trichrome staining were performed. Compared with NC mice, DKD mice showed mesangial cell proliferation, mesangial matrix expansion, and glycogen deposition on H&E and PAS staining. In contrast, TD treatment significantly improved the diabetes-induced glomerular histopathology. These morphological improvements suggest that TD suppresses mesangial matrix accumulation, a hallmark of diabetic glomerulosclerosis. Meanwhile, Masson trichrome staining showed that TD treatment attenuated interstitial fibrosis in DKD mice (Fig. 5N). In summary, these results indicate that TD exerts a potent renoprotective effect.

#### TD attenuated oxidative stress induced renal injury in DKD mice

**Fig. 6:**
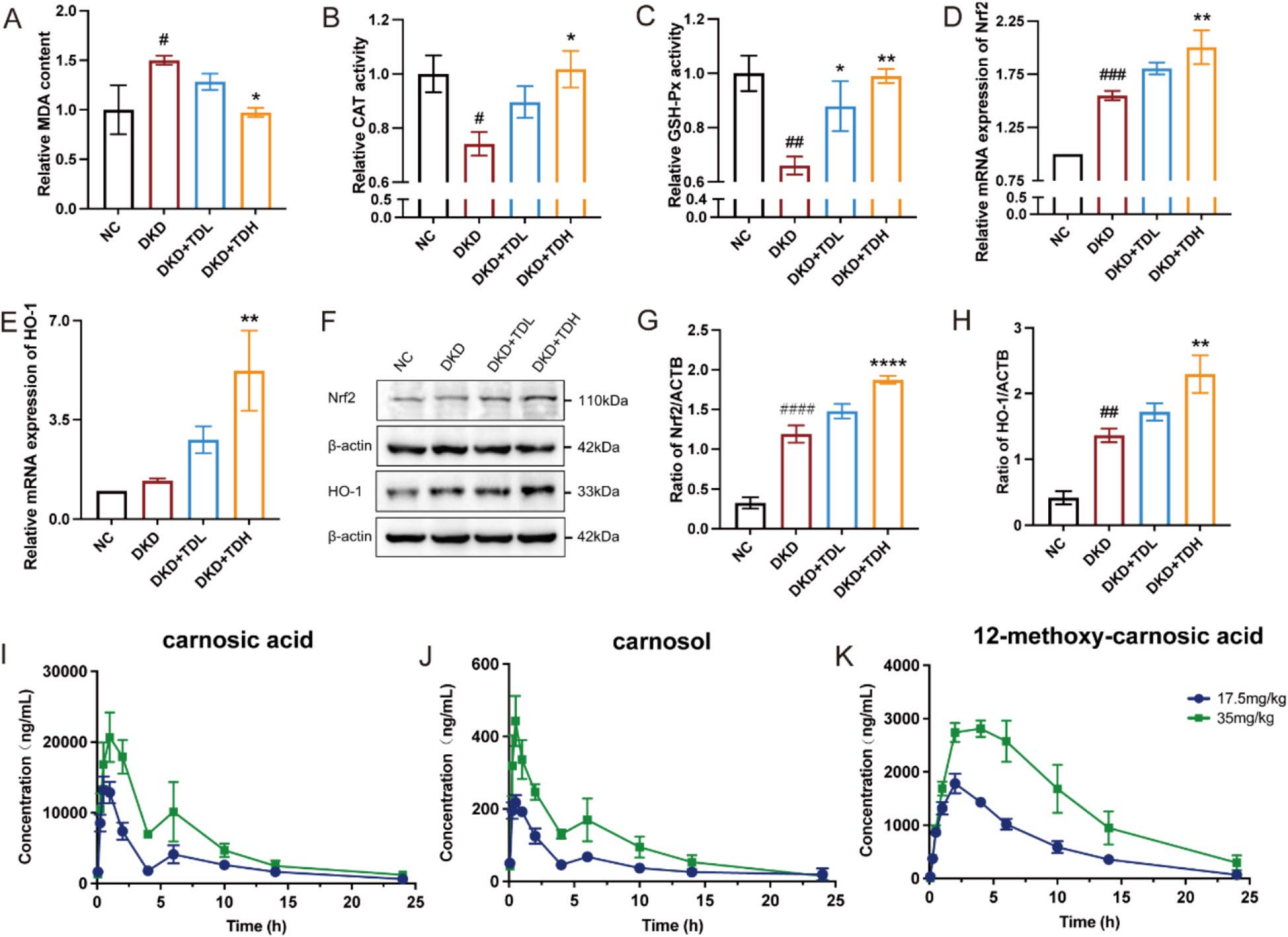
Investigation of therapeutic effects of TD on renal oxidative stress in DKD mice. (*n* = 6). **A** The relative content of MDA. **B**-**C** The relative activity of CAT (**B**) and GSH-Px (**C**). **D**, **E** The relative mRNA expression of Nrf2 (**D**) and HO-1 (**E**) in mouse kidney. **F–H** The protein expression levels of Nrf2 **(G)** and HO-1 **(H)** were detected by western blot and quantification with β-actin as control. **I**–**K** Concentration–time curves of carnosic acid (**I**), carnosol (**J**), and 12-methoxy-carnosic acid (**K**) in rat plasma after oral administration of TD. Data were shown as Mean ± SEM. Statistical analysis was performed using one-way ANOVA, ^*^P < 0.05, ^**/##^*P* < 0.01, ^***/###^*P* < 0.001, ^****/####^*P* < 0.0001. ^#^denotes comparison with NC group, and ^*^ denotes comparison with model group.

Given that TD is a potent antioxidant and oxidative stress is a key pathological hallmark of DKD [15], we investigated whether TD could relieve renal oxidative stress in DKD mice model. As shown in Fig. 6A–C, TD reduced renal levels of malondialdehyde (MDA) and increased the activities of superoxide dismutase (SOD) and glutathione peroxidase (GSH-Px) in DKD mice kidney. These results suggest that TD attenuated renal oxidative stress in DKD mice.

Additionally, Nrf2 is a key regulator of the cellular antioxidant response. HO-1 is the downstream target gene of Nrf2 and exerts antioxidant, and cytoprotective effects through the coordinated actions of its enzymatic products [16]. To detect whether TD activates Nrf2/HO-1 pathway, we evaluated the mRNA and proteins expression levels of Nrf2 and HO-1 in mouse kidney tissue. As shown in Fig. 6D–E, TD treatment significantly increased the mRNA level of Nrf2 and HO-1 in a dose-dependent manner. Western blotting results further revealed that the protein levels of Nrf2 and HO-1 were also elevated following TD treatment (Fig. 6F–H). Taken together, these results indicate that TD exhibited potential in vivo antioxidation effects by activation of Nrf2/HO-1 pathway.

#### In vivo absorption profile of the TD

As shown in Fig. S3, the major components (carnosic acid, carnosol, and 12-methoxy-carnosic acid) were identified in rat plasma following intragastric administration (i.g.), and the concentration–time curves are shown in Fig. 6I–K. The double-peak plasma concentration–time profiles of carnosic acid and carnosol suggest possible enterohepatic circulation. As shown in Table. S9, although carnosic acid readily degrades to carnosol in vitro, their ${\mathrm{T}}_{\mathrm{max}}$ values indicate no obvious interconversion in vivo. The oral bioavailability of carnosic acid, carnosol, and 12-methoxy-carnosic acid was 43.2%, 24.4%, and 91.8%, respectively, demonstrating that the TD major components maintain good bioavailability. In addition, the validation of an LC–MS/MS method for the analysis of blood samples was shown in Table S10–13.

To investigate the in vivo toxicity of TD, we have performed acute toxicity study. The results demonstrated that TD has an LD₅₀ of 3.721 g/kg (95% CI 2.933–5.805 g/kg) (Table S14). In addition, no obvious toxicity was detected in the major organs of surviving mice (Fig. S4A), as confirmed by histological analysis using H&E staining (Fig. S4B). Necropsy revealed mild intestinal distension in some deceased mice, and histological analysis showed potential damage to the small intestinal villi. The no observed adverse effect level (1.20 g/kg) is much higher than the effective dose (50 mg/kg).

## Discussion

DKD is one of the fastest-growing causes of chronic kidney disease, with about 40% of diabetic patients progressing to DKD. It has been recognized as a major cause of end-stage renal disease (ESRD) [17]. DKD is initially driven by metabolic disorders, including hyperglycemia, hyperlipidemia, and insulin resistance. These issues then trigger a cascade of pathological events, such as glomerular hyperfiltration, inflammation, oxidative stress, and progressive fibrosis, eventually leading to loss of renal function [18, 19]. Rosemary is rich in bioactive compounds that have been shown to regulate glucolipid metabolism and mitigate oxidative stress. That makes rosemary a promising therapeutic candidate for the invention of DKD.

In this study, UPLC-Q/TOF–MS/MS was used to elucidate the pharmacodynamic material basis of rosemary leaves. We characterized 38 components, including 13 diterpenoids, 6 phenolic acids, 12 flavonoids, and 4 triterpenoids. In vitro screening using a cellular DKD model identified diterpenoids as the main active components contributing to its anti-DKD effects.

Recent research of rosemary diterpenoids extraction primarily focused on carnosic acid. Conventional extraction methods mainly rely on Soxhlet, maceration, and infusion. Grzegorz et. al. developed an ultrasound-related maceration method, yielding a carnosic acid content of 147.5 mg/g [20]. Salamatin et. al used Soxhlet extraction to extract diterpenoids from leaves of *Salvia officinalis*, achieving a total diterpenoids content of 50 mg/g [21]. However, Soxhlet extraction needs heating, which brings a risk of bioactive constituent degradation. Maceration is time-consuming and often uses toxic organic solvents such as methanol, acetone, ether, and hexane [22]. To address the limitation of conventional methods, some new extraction methods have been established. Wang et. al. reported a method that combined ultrasound-assisted extraction with high-speed countercurrent chromatography separation based on hydrophobic deep eutectic solvent. Although the approach reduces the use of organic solvents, it remains challenging in industrial application, and the content of carnosic acid is low (30%) [23]. Mo et.al extracted carnosic acid using an in-house column and achieving a purity over 90% [24]. However, the reliance on a laboratory-made chromatographic column limited its industrial applications. Thibault et. al. developed an online extraction-supercritical fluid chromatography method that enables extraction at 25 °C, which reduced carnosic acid degradation. This method obtained a product containing 49% carnosic acid [25]. Compared with reported methods, our enrichment method is environmentally friendly with only water and ethanol. The additional vitamin C stopped degradation of diterpenoids during extraction, and vitamin C can be easily removed from the final product. In summary, this method is simple, yielding a final product containing 60% carnosic acid and 80% total diterterpenoids. While the method is efficient and environmentally friendly, the current TD yield is 40%, suggests that the utilization of rosemary leaves could be further improved. One future direction is to further optimize the extraction process to improve the yield of TD.

To evaluate the therapeutic effects of the TD extract on DKD, we established a DKD mouse model. After ten weeks of treatment, TD not only ameliorated disorders of glucose and lipid metabolism, but also mitigated renal injury. In consideration of renal oxidative stress injury is a hallmark of DKD, we assessed key oxidative stress related factors subsequently. The results showed that TD decreased MDA level and improved SOD and GSH-Px activity, suggesting that TD attenuated oxidative stress in the DKD mice kidney. Additionally, TD treatment activated Nrf2/HO-1 pathway by increasing the expression of mRNA and protein levels of Nrf2 and HO-1, indicating the potential in vivo antioxidation activity.

To further explore the in vivo absorption of TD, pharmacokinetic analyses of its major components were conducted. The results showed the major components of TD possessed good bioavailability, which supports its potential application in the treatment of DKD.

## Conclusion

This study developed a novel method for enrichment of rosemary diterpenoids, providing a practical framework for enrichment of other degradable natural products. The enriched diterpenoid extract demonstrated potent renoprotective effects in DKD mice, indicating its potential as a therapy in the treatment of DKD.

## Supplementary Information

Supplementary Material 1.

## Data Availability

All data generated during this study are included in this published article and its supplementary information files.

## Abbreviations

DKD: Diabetic kidney disease
TD: Total diterpenoids
RE: Rosemary leaves extract
TG: Total triacylglycerol
TC: Total cholesterol
BUN: Blood urea nitrogen
LDL-C: Low-density lipoprotein cholesterol
HDL-C: High-density lipoprotein cholesterol
ITT: Insulin tolerance test
OGTT: Oral glucose tolerance test
AUC: Area under the curve
H&E: Hematoxylin and eosin
PAS: Periodic acid–Schiff
HG: High glucose
PA: Palmitic acid
Dap: Dapagliflozin
UAE: Urinary albumin excretion
UACR: Urine microalbumin-to-creatinine ratio
ESRD: End-stage renal disease
