# Supplementary material for: A stabilized enrichment method for rosemary diterpenoids and their therapeutic potential in diabetic kidney disease
Source: Chin Med. 2026 May 6;21:127. doi: 10.1186/s13020-026-01404-1 (PMC13147855; doi:10.1186/s13020-026-01404-1)
Supplement: Supplementary file 1 — Supplementary Material 1. [file 13020_2026_1404_MOESM1_ESM.docx]

**A Stabilized Enrichment Method for Rosemary Diterpenoids and Their Therapeutic Potential in Diabetic Kidney Disease**

Jiali Wei, Qian Xiao, Hua Yang, Fei Li^*^, Ping Li^*^

*State Key Laboratory of Natural Medicines, China Pharmaceutical University, Nanjing, 211198, China*

^*^Corresponding authors

E-mail address: lifeicpu@163.com (F. Li), liping2004@126.com (P. Li)

**Table S1.** Orthogonal experimental factors and levels

**Table S2**. Results of the orthogonal experiment.

**Table S3**. Analysis of variance table of the orthogonal experiment.

**Table S4**. Linearity range and standard curves of reference standards.

**Table S5.** Comparation of different variables in single factor experiment of extraction optimization.

**Table S6.** Coefficients of determination (*R*^2^) of the linear fitting for the thermal degradation kinetics of carnosic acid at different temperatures.

**Table S7.** The RT-qPCR primer sequences used in this study.

**Table S8**. Peak assignments of degradable compounds and degradation products.

**Table S9.** Pharmacokinetic parameters of the three analytes in SD rats.

**Table S10.** The regression equation, correlation coefficient, linear range and LLOQs of three compounds.

**Table S11.** Precision and accuracy for the analytes in rat plasma.

**Table S12.** Extraction recovery and matrix effect data for the analytes in rat plasma.

**Table S13.** Stability of the analytes in rat plasma.

**Table S14**. Number of deaths in mice following a single administration at various doses.

**Fig. S1.** **A** Total ion chromatograms of different fractions from rosemary. **B-E** Cell viability was evaluated using the CCK-8 assay after 24-h treatment with different concentrations of extracts.

**Fig. S2**. Stability improvement of TD

**Fig. S3.** Analysis of rat plasma samples.

**Fig. S4.** The acute toxicity study of TD.

**Method**

1. Quantitative degradation kinetics of carnosic acid

2. Preparation of plasma samples

3. Analysis of plasma samples

4. Validation of an LC-MS/MS method for the analysis of blood samples

**Table S1.** Orthogonal experimental factors and levels

| **Level** | **A: washing method** | **B: solid/liquid ratio（g/mL）** | **C: number of cycles** |
| --- | --- | --- | --- |
| 1 | ultrasonication | 1:2 | 2 |
| 2 | grind | 1:3 | 3 |
| 3 | shaking | 1:4 | 4 |

**Table S2**. Results of the orthogonal experiment.

| **Number** |  | **A** | **B** | **C** | **Area of rosmarinic acid** |
| --- | --- | --- | --- | --- | --- |
| 1 |  | 1 | 1 | 1 | 1131.378 |
| 2 |  | 1 | 2 | 3 | 1165.350 |
| 3 |  | 1 | 3 | 2 | 1193.967 |
| 4 |  | 2 | 1 | 3 | 713.218 |
| 5 |  | 2 | 2 | 2 | 737.882 |
| 6  7  8  9 | K1  K2  K3  r | 2  3  3  3  1163.56  706.24  698.74  464.83 | 3  1  2  3  836.69  867.25  864.60  30.56 | 1  3  1  2  832.51  888.02  848.02  55.51 | 667.619  665.483  698.524  732.201 |

**Table S3**. Analysis of variance table of the orthogonal experiment.

| **Factor** | **Deviation sums of squares** | **Degree of freedom** | **F**  **ratio** | **F**  **Threshold** | **P**  **value** | **significance** |
| --- | --- | --- | --- | --- | --- | --- |
| A | 425268.765 | 2 | 212634.382 | 339.384 | 0.003 | ******* |
| B | 555.755 | 2 | 277.878 | 0.444 | 0.693 |  |
| C | 3758.145 | 2 | 1879.072 | 2.999 | 0.250 |  |
| Error | 1253.062 | 2 | 626.531 |  |  |  |

**Table S4**. Linearity range and standard curves of reference standards.

|  | **component** | **linearity range**  **(μg/mL)** | **standard curve** | ***r*** | **Relative**  **content** |
| --- | --- | --- | --- | --- | --- |
| 1 | apigenin | 0.06~0.90 | y = 9.6462x - 0.2103 | 0.9995 | 0.03% |
| 2 | hispidulin | 2.74~16.46 | y = 13.384x - 1.6825 | 0.9998 | 0.23% |
| 3 | epirosmanol | 10.80~108.00 | y = 1.7855x - 6.8074 | 0.9958 | 12.60% |
| 4 | rosmanol | 0.35~5.63 | y = 0.7151x - 0.0233 | 0.9998 | 0.49% |
| 5 | genkwanin | 2.50~15.03 | y = 6.1292x + 0.4069 | 0.9998 | 0.58% |
| 6 | pectolinarigenin | 0.07~1.09 | y = 10.255x + 0.8458 | 0.9999 | 0.05% |
| 7  8 | carnosol  carnosic acid | 4.06~129.91  9.18~458.90 | y = 1.8824x + 2.0559  y = 1.3330x - 1.7818 | 0.9995  0.9999 | 6.43%  59.11% |
| 9 | 12-methoxy-carnosic acid | 0.90~9.04 | y = 1.0512x + 0.0284 | 0.9992 | 3.17% |
| 10 | betulinic acid | 3.49~55.86 | y = 1.8687x - 1.7708 | 0.9998 | 2.25% |

**Table S5.** Comparation of different variables in single factor experiment of extraction optimization.

|  | **Factor** | **Different variables** | | | | | | | | | | | |
| --- | --- | --- | --- | --- | --- | --- | --- | --- | --- | --- | --- | --- | --- |
| **Extraction**  **of TD** | **Method** | Cold maceration | | | | Ultrasonication | | | | Heat reflux | | | |
|  |  | *Ref* | | | | ** | | | | **** | | | |
|  | **Ethanol concentration** | 75% | | | | 85% | | | | 95% | | | |
|  |  | *Ref* | | | | ** | | | | *** | | | |
|  | **solid/liquid ratio** | 1:5 | | | 1:8 | | | 1:10 | | | 1:12 | | |
|  |  | *Ref* | | | * | | | ** | | | ** | | |
|  | **Temperature (℃)** | 80 | | | | 90 | | | | 100 | | | |
|  |  | *Ref* | | | | ns | | | | ns | | | |
|  | **Time (h)** | 0.5 | | | 1.0 | | | 1.5 | | | 2.0 | | |
|  |  | *Ref* | | | * | | | * | | | * | | |
|  |  |  | | | *Ref* | | | ns | | | ns | | |
|  | **Cycles** | 1 | | | 2 | | | 3 | | | 4 | | |
|  |  | *Ref* | | | ns | | | * | | | * | | |
| **Removal**  **of**  **phenolic acid** | **Washing method** | grinding | | shaking | | | Ultrasonication(W) | | | | | | |
|  |  |  |  |  |  |  | 40 | | 60 | | | 80 | |
|  |  | *Ref* | | *** | | | **** | | **** | | | **** | |
|  |  |  | |  | | | *R*ef | | ns | | | ns | |
|  | **Washing solid/liquid ratio** | 1:2 | 1:3 | | | 1:4 | | 1:5 | | 1:6 | | | 1:7 |
|  |  | *Ref* | **** | | | **** | | **** | | **** | | | **** |
|  |  |  |  | | | *Ref* | | ns | | ns | | | ns |
|  | **cycles** | 1 | | 2 | | | 3 | | 4 | | | 5 | |
|  |  | *Ref* | | **** | | | **** | | **** | | | **** | |
|  |  |  | | *Ref* | | | ns | | ns | | | ns | |

*Ref*：control group

“^*^” denotes comparison with *Ref*. Statistical analysis of different variables was performed using one-way anova. ^*^*P* < 0.05, ^**^*P* < 0.01, ^***^*P* < 0.001, ^****^*P* < 0.0001. ns：no significance.

**Table S6.** Coefficients of determination (*R*^2^) of the linear fitting for the thermal degradation kinetics of carnosic acid at different temperatures.

| Temperature (℃) | *R^2^* | | |  |
| --- | --- | --- | --- | --- |
|  | Zero-order | First-order | Second-order | |
| 4 | 0.9348 | 0.9343 | 0.9339 | |
| 25 | 0.9812 | 0.9774 | 0.9733 | |
| 40 | 0.9919 | 0.9842 | 0.9738 | |
| 50 | 0.9943 | 0.9719 | 0.9307 | |
| 60 | 0.9926 | 0.9235 | 0.792 | |

**Table S7.** The RT-qPCR primer sequences used in this study.

|  | **Primer name** | **Base sequence (5' to 3')** |
| --- | --- | --- |
| HK2 cell | ACTB-F | CTAATGGTGGAAACCCACAACG |
|  | ACTB-R | TATCGCCAGGAATTGTTGCTG |
|  | COL1A1-F | GAGGGCCAAGACGAAGACATC |
|  | COL1A1-R | CAGATCACGTCATCGCACAAC |
|  | FN-F | GAGAATAAGCTGTACCATCGCAA |
|  | FN-R | CGACCACATAGGAAGTCCCAG |
|  | KIM-1-F | TCACATCCATGTGCTGGAAT |
|  | KIM-1-R | CGTGTGTCCTTCCGATAGGT |
|  | α-SMA-F | AAAAGACAGCTACGTGGGTGA |
|  | α-SMA-R | GCCATGTTCTATCGGGTACTTC |
| Mouse kidney | ACTB-F | GTGGGAATGGGTCAGAAGGA |
|  | ACTB-R | GGCTGGGGTGTTGAAGGTC |
|  | NRF2-F | CAGCATAGAGCAGGACATGGAG |
|  | NRF2-R | GAACAGCGGTAGTATCAGCCAG |
|  | HO-1-F | CACTCTGGAGATGACACCTGAG |
|  | HO-1-R | GTGTTCCTCTGTCAGCATCACC |

**Table S8**. Peak assignments of degradable compounds and degradation products.

| **Peak** | **t_R_ (min)** | **identification** | **[M-H]^-^ (m/z)** |
| --- | --- | --- | --- |
| 1**^#^** | 9.863 | epirosmanol | 345.1688 |
| 2* | 10.363 | rosmanol | 345.1689 |
| 3* | 10.912 | epiisorosmanol | 345.1686 |
| 4* | 13.789 | carnosol | 329.1691 |
| 5* | 14.212 | carnosic acid quinone | 329.1729 |
| 6* | 14.467 | 11-ethoxyrosmanol semiquinone | 373.1965 |
| 7* | 14.906 | epirosmanol ethy lether | 373.1964 |
| 8* | 15.572 | unknown | 333.1632 |
| 9**^#^** | 16.178 | carnosic acid | 331.1922 |

**^#^**degradable compounds，*degradation products

**Table S9.** Pharmacokinetic parameters of the three analytes in SD rats (*n* = 6).

|  | Group | Tmax  (h) | Cmax  (ng·mL^-1^) | T1/2  (h) | AUC(0-t)  (ng·h^-1^·mL^-1^) | AUC(0-∞)  (ng·h^-1^·mL^-1^) | MRT(0-t)  (h) | MRT(0-∞)  (h) | Bioavailability  (%) |
| --- | --- | --- | --- | --- | --- | --- | --- | --- | --- |
| Carnosic acid | TDL | 0.75±0.27 | 13947.26±4267.80 | 8.47±4.08 | 68478.22±23787.95 | 75434.93±23735.14 | 6.69±0.75 | 9.95±1.66 | 43.10 |
|  | TDH | 1.25±0.61 | 22485.06±6355.40 | 6.78±3.47 | 137542.14±61187.64 | 152504.96±86016.38 | 6.20±1.22 | 8.54±3.36 | 43.30 |
| Carnosol | TDL | 0.62±0.31 | 225.96±47.18 | 7.38±6.34 | 1030.38±296.26 | 1314.99±668.96 | 5.32±1.20 | 10.48±7.85 | 23.80 |
|  | TDH | 0.71±0.64 | 470.03±149.39 | 6.80±3.49 | 2394.30±1097.54 | 2698.00±1379.14 | 5.93±1.41 | 7.00±2.14 | 24.90 |
| 12-methoxy-carnosic acid | TDL | 2.33±0.82 | 1791.89±442.34 | 4.34±1.34 | 15161.00±3508.4 | 15686.38±3938.94 | 6.63±1.12 | 7.35±1.72 | 86.50 |
|  | TDH | 5.00±3.03 | 3295.85±558.47 | 5.50±3.19 | 33995.83±13185.01 | 37979.66±17932.6 | 7.56±1.69 | 9.06±3.20 | 97.00 |

**Table S10.** The regression equation, correlation coefficient, linear range and LLOQs of three compounds.

| Compound | Equation | r | Linear range  (ng/mL) | LLOQ  (ng/mL) |
| --- | --- | --- | --- | --- |
| Carnosic acid | y = 0.01055x + 0.3780 | 0.9999 | 21.4~42856.9 | 21.4 |
| Carnosol | y = 0.02190x + 1.1378 | 0.9996 | 4.4~1097.6 | 4.4 |
| 12-methoxy-carnosic acid | y=0.005033x+0.004731 | 0.9999 | 19.3~5777.7 | 19.3 |

**Table S11.** Precision and accuracy for the analytes in rat plasma (*n* = 6).

| Compound | Concentration  (ng/mL) | Intra-day | | Inter-day | |
| --- | --- | --- | --- | --- | --- |
|  |  | RE% | RSD% | RE% | RSD% |
| Carnosic acid | 64.29 | -3.33 | 10.61 | 7.33 | 9.80 |
|  | 17142.76 | 9.67 | 3.29 | 10.76 | 3.29 |
|  | 34285.52 | 14.02 | 1.06 | 13.92 | 1.06 |
| Carnosol | 16.46 | -6.83 | 6.21 | -6.50 | 8.95 |
|  | 878.08 | -7.32 | 1.65 | -7.58 | 1.65 |
|  | 1756.16 | -8.88 | 1.83 | -11.11 | 1.83 |
| 12-methoxy-carnosic acid | 173.33 | 14.80 | 5.32 | 14.23 | 3.78 |
|  | 2311.08 | -8.50 | 1.31 | -8.28 | 1.31 |
|  | 4622.15 | -12.25 | 1.65 | -12.18 | 1.65 |

**Table S12.** Extraction recovery and matrix effect data for the analytes in rat plasma (*n* = 6).

| Compound | Concentration (ng/mL) | Mean (%) | RSD (%) |
| --- | --- | --- | --- |
| Carnosic acid | 64.29 | 102.39 | 7.34 |
|  | 17142.76 | 105.39 | 3.41 |
|  | 34285.52 | 114.03 | 2.32 |
| Carnosol | 16.46 | 93.85 | 8.34 |
|  | 878.08 | 86.62 | 5.37 |
|  | 1756.16 | 87.32 | 5.73 |
| 12-methoxy-carnosic acid | 173.33 | 111.43 | 3.12 |
|  | 2311.08 | 110.72 | 2.90 |
|  | 4622.15 | 112.51 | 2.78 |

**Table S13.** Stability of the analytes in rat plasma (*n* = 6).

| Compound | Concentration (ng/mL) | Mean (%) | RSD (%) |
| --- | --- | --- | --- |
| Carnosic acid | 64.29 | 106.20 | 11.47 |
|  | 17142.76 | 111.05 | 3.15 |
|  | 34285.52 | 113.52 | 0.93 |
| Carnosol | 16.46 | 91.18 | 6.40 |
|  | 878.08 | 91.84 | 3.24 |
|  | 1756.16 | 89.65 | 2.42 |
| 12-methoxy-carnosic acid | 173.33 | 116.78 | 3.78 |
|  | 2311.08 | 92.02 | 2.24 |
|  | 4622.15 | 87.94 | 1.93 |

**Table S14**. Number of deaths in mice following a single administration at various doses.

| Group | Dose | Number of deaths |
| --- | --- | --- |
| NC | 0.5%CMC-Na | 0 |
| TD-1 | 5.00 g/kg TD | 7 |
| TD-2 | 3.50 g/kg TD | 4 |
| TD-3 | 2.45 g/kg TD | 3 |
| TD-4 | 1.71 g/kg TD | 1 |
| TD5 | 1.20 g/kg TD | 0 |


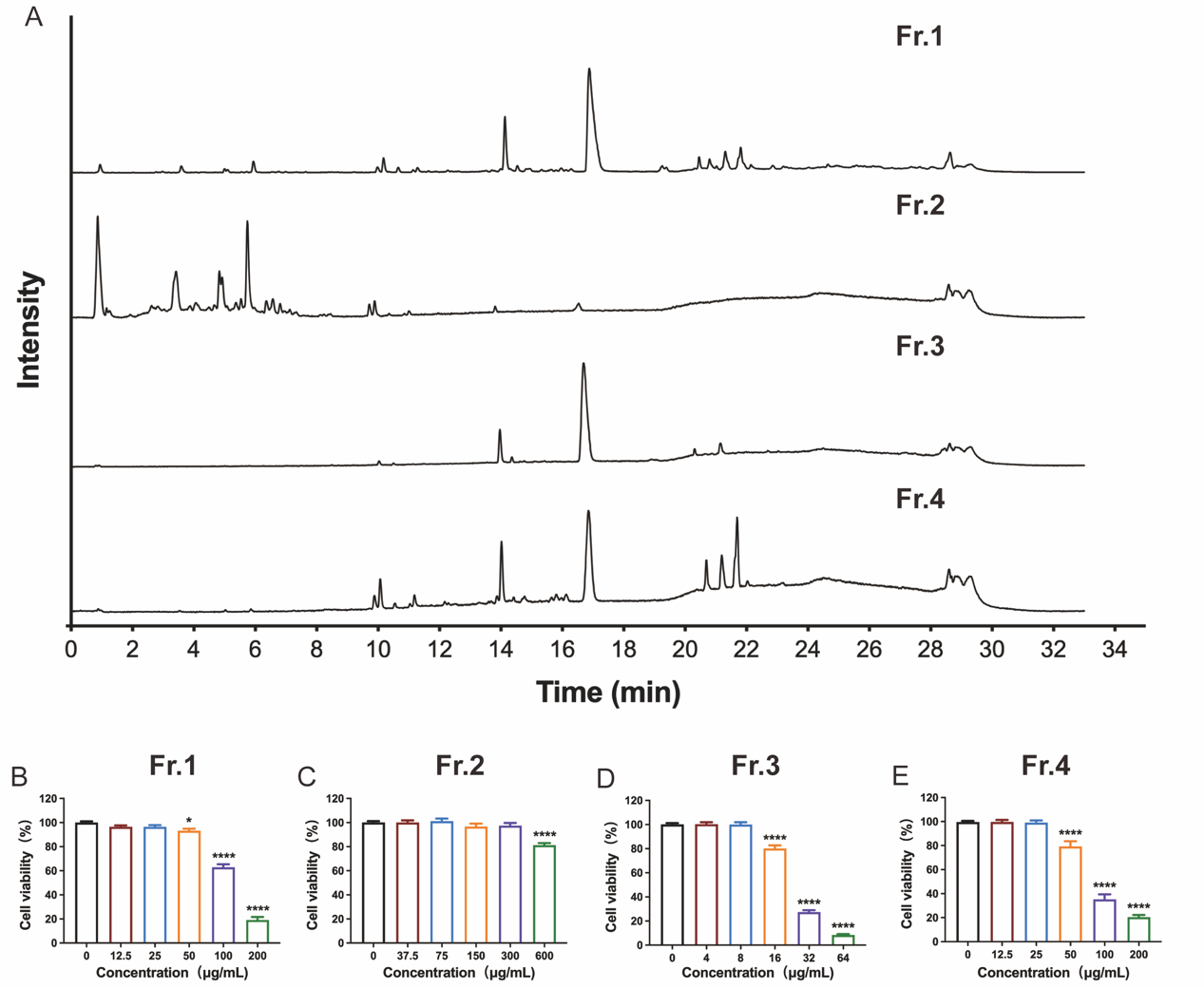


**Fig. S1.** **A** Total ion chromatograms of different fractions from rosemary. **B-E** Cell viability was evaluated using the CCK-8 assay after 24-h treatment with different concentrations of extracts. ^*^*P* < 0.05, ^**^*P* < 0.01, ^***^*P* < 0.001, ^****^*P* < 0.0001. “^*^” denotes comparison with model group.


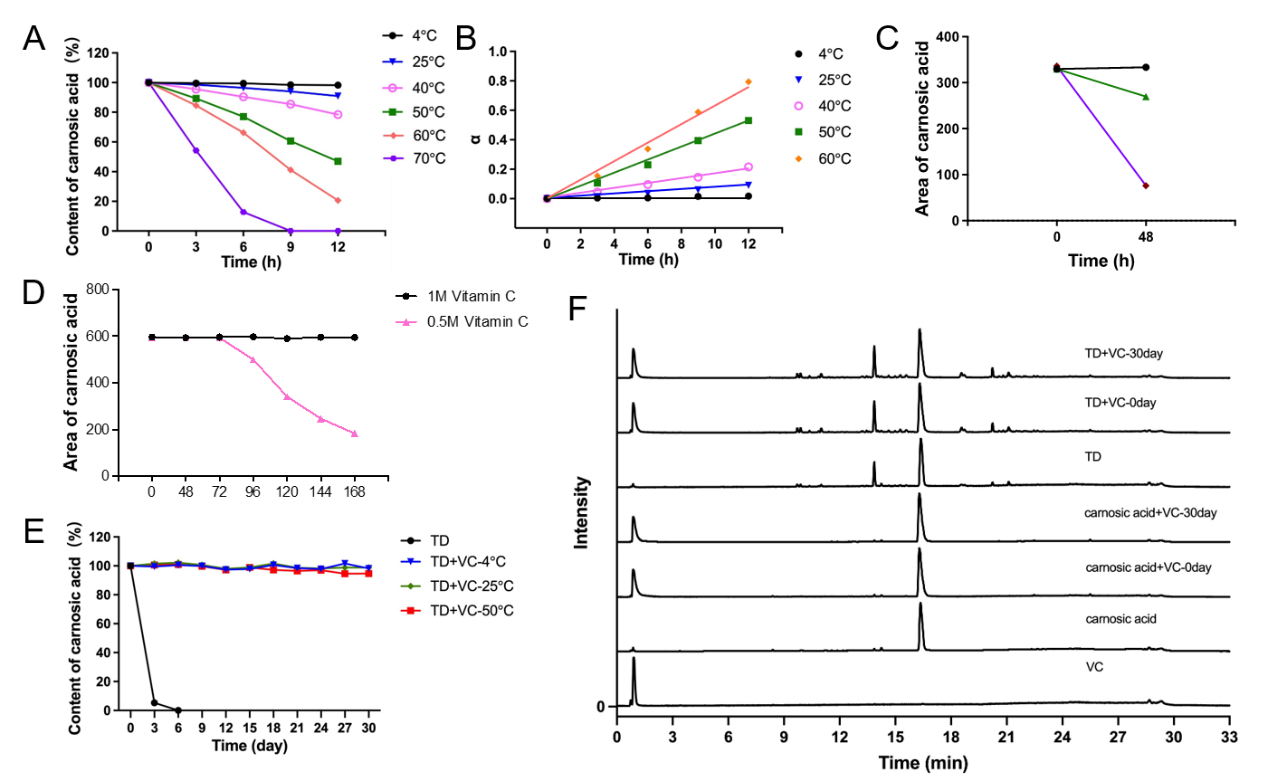


**Fig. S2**. Stability improvement of TD. (**A**) Thermal stability of the carnosic acid. (**B**) Thermal degradation kinetic curves of carnosic acid, **α** represents carnosic acid conversion rate. (**C**) Influence of different additives on carnosic acid stability. **(D)** Optimization of vitamin C concentration for carnosic acid stability. (**E**) Evaluation of carnosic acid stability in TD solution containing vitamin C over one month at 4, 25, and 50 ºC. (**F**) Chromatography of carnosic acid/TD ± vitamin C.

**
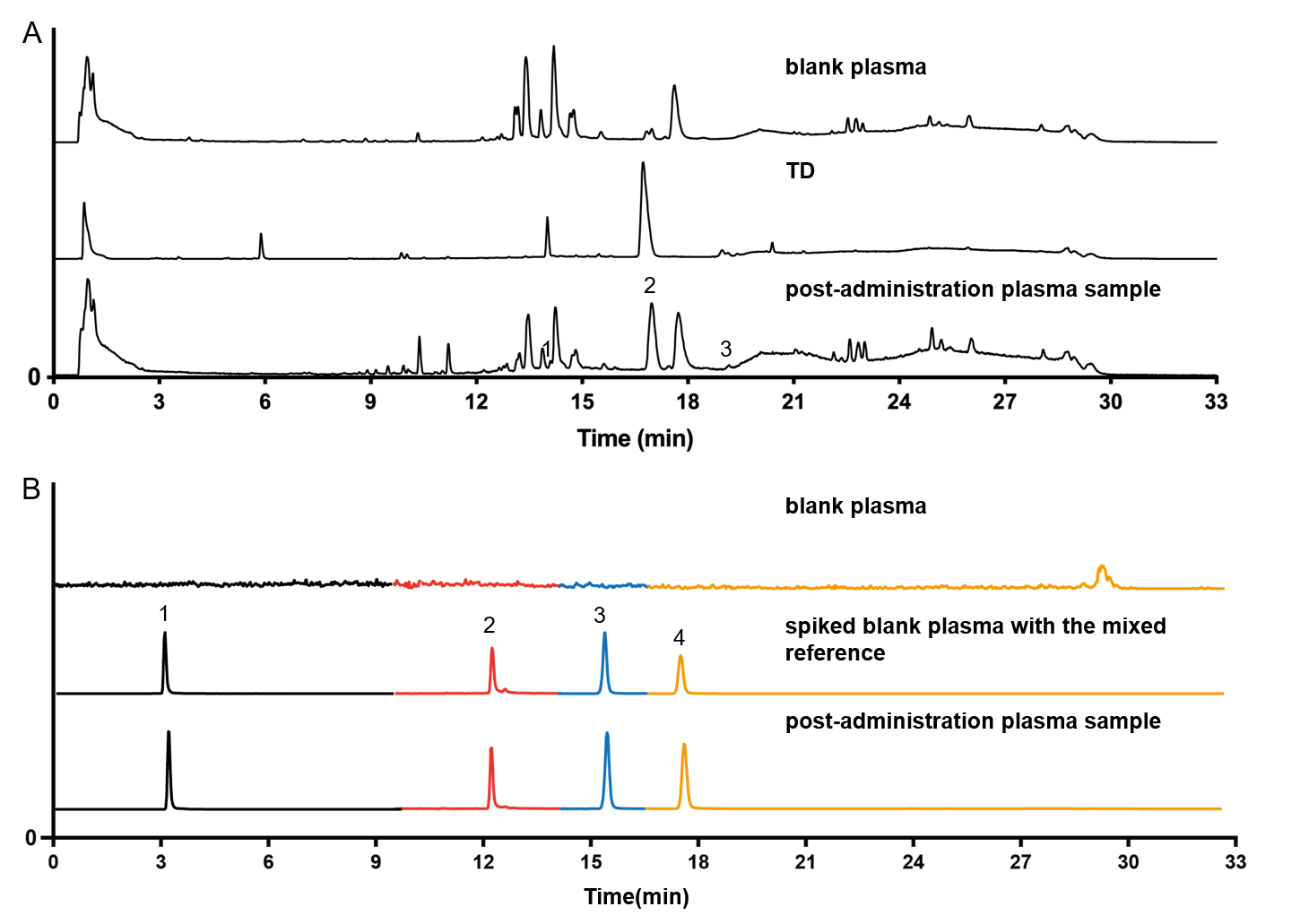
Fig. S3.** Analysis of rat plasma samples. **A** UPLC-Q/TOF-MS analysis of rat blank plasma, TD, and post-administration plasma sample. 1-carnosol, 2-carnosic acid, 3-12-methoxy-carnosic acid. **B** UPLC-QQQ-MS analysis of rat blank plasma, spiked blank plasma with the mixed reference, and post-administration plasma sample. 1-bergenin, 2-carnosol, 3-carnosic acid, 4-12-methoxy-carnosic acid.


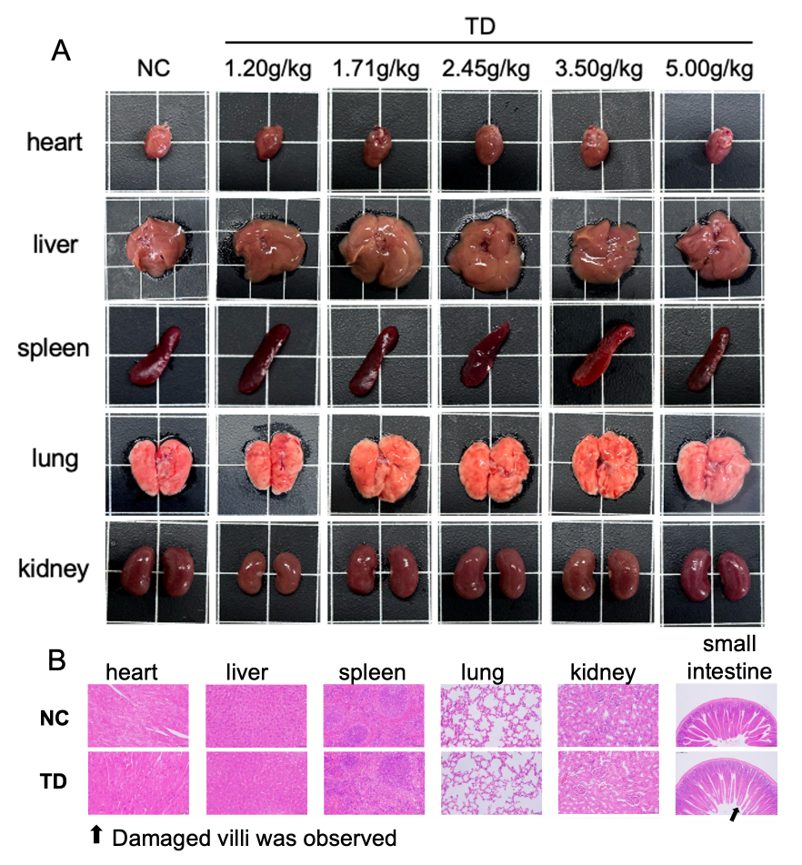


**Fig. S4.** The acute toxicity study of TD. **A** The representative images of major organs from surviving mice in NC and treatment groups. **B** H&E staining analysis of major organs in NC group and TD treatment group (5 g/kg). Similar results were observed in at least three mice (*n* = 5).

**Supplementary Methods**

**1.Quantitative degradation kinetics of carnosic acid**

**1.1 Heat treatment**

For each heating experiment, 10.00 mg of carnosic acid powder was weighed into 100 mL corked conical flasks, respectively and dissolved in 50% ethanol. The samples were heated at different temperatures (4, 25, 40, 50, 60, and 70 ºC). The concentration of carnosic acid in the test solution was measured at different time points (0, 3, 6, 9, and 12 h). Each temperature and time condition were performed in triplicate, and the mean value was reported.

**1.2 Kinetic calculation**

First, the carnosic acid conversion rate (*α*) was computed from Eq. (1) by utilizing the carnosic acid contents:

*α (%)* = (C_0_-C_t_)/C_0_×100 (1)

where *C*_0_ is the initial concentration of carnosic acid in the untreated sample and *C*t is the residual concentration measured after heating at a given temperature for a designated time ‘t’.

Subsequently, the thermal degradation of carnosic acid was interpreted within the framework of chemical kinetics and fitted using zero-order, first-order and second-order kinetic model.

Zero-order kinetic model: *α*×*C_0_=kt* (2)

First-order kinetic model: *-ln (1-α) = kt* (3)

Second-order kinetic model:*C_0_*×kt+1=1/(1-*α)* (4)

**2. Preparation of** **plasma samples**

SD rats (8 weeks，half male and half female) were purchased from Charles River Laboratory (Pinghu, China). All mice were housed in SPF conditions on a standard 12-h light-dark cycle with free access to food and water at a controlled temperature (25 ℃ ± 2 ℃).

The SD rats were randomly divided into 3 groups (n = 6 per group; 3 males and 3 females): (i) TDL group (17.5 mg/kg TD, gavage); (ii) TDH group (35 mg/kg TD, gavage); (iii) TDV group (5 mg/kg TD, intravenous injection). Following a 12 h fasting period with free access to water, blood was collected via orbital venous plexus at 0, 0.083, 0.25, 0.5, 1, 2, 4, 6, 10, 14, and 24 h after single administration. Blood samples were collected in heparin-coated tubes and centrifuged at 4000 rpm for 10 min to collect plasma. Subsequently, plasma aliquots (50 μL) were mixed with 1000 μL methanol (containing 0.1% vitamin C) and vortexed for 1 min, followed by centrifugation at 12,000 rpm for 10 min. The supernatant was transferred to microcentrifuge tubes, dried under a nitrogen stream, and then reconstituted with 1000 μL methanol. The mixture was centrifuged at 12,000 rpm for 10 min, and the resulting supernatant was ready to analysis. Bergenin was added as internal standard (IS) at 300 ng/mL.

**3. Analysis of plasma samples**

The plasma samples were analyzed using a Shimadzu LC-20A UPLC system. Chromatographic conditions were same as those described in section 3.5.1, and the injection colume was 2 μL. Mass spectrometric analysis was performed using a Shimadzu 8050 triple quadrupole mass spectrometer equipped with an electrospray ionization (ESI) source. The MS parameters were configured as follows: nebulizing gas flow, 3 L/min; heating gas flow, 10 L/min; drying gas flow, 10 L/min; interface voltage, +4.0 kV/−3.0 kV; interface temperature, 300 ℃; heating block temperature, 400 ℃; DL temperature, 250 ℃; collision gas pressure, 270 kPa. The mass spectrometric analysis parameters are shown as **Table S15.**

**Table S15**. Summary of target analytes and corresponding MRM parameters.

| Compound | Precursor ion | Product ion | Q1 Pre Bias(V) | CE | Q3 Pre Bias(V) |
| --- | --- | --- | --- | --- | --- |
| Begenin | 327.10 | 249.20 | 12.0 | 15.0 | 11.0 |
| Carnosic acid | 331.20 | 244.50 | 12.0 | 21.0 | 13.0 |
| Carnosol | 329.20 | 285.25 | 12.0 | 21.0 | 13.0 |
| 12-methoxy-carnosic acid | 345.30 | 286.50 | 10.0 | 26.0 | 13.0 |

**4. Validation of an LC-MS/MS method for the analysis of blood samples**

**4.1. Specificity**

Specificity was evaluated by analysis of blank plasma, spiked blank plasma with the mixed reference standards and IS, and post-administration plasma samples.

**4.2. Linearity and LLOQ**

Calibration samples were prepared by spiking blank plasma with mixed working solutions and the IS solution. Calibration curves were constructed by plotting the peak area ratios (y, analyte/IS) versus nominal concentrations (x) using weighted least squares regression.

**4.3. Precision and accuracy**

LQC, MQC, and HQC samples (n = 6) were employed to evaluate intra-day and inter-day precision and accuracy. Accuracy was determined as percentage relative error (%RE), while precision was calculated as percentage relative standard deviation (%RSD). Acceptance criteria were defined as ≤ 15% for precision and accuracy across all tested concentrations.

**4.4. Recovery and matrix effects**

LQC, MQC, and HQC samples (n = 6) were employed to assess extraction recovery and matrix effects. The recovery rate was determined by comparing the peak area ratios of analytes in the extracted samples with those of post-extraction spiked samples. Matrix effects were evaluated by comparing the peak area ratios of analytes in extracted samples with those in neat standard solutions.

**4.5. Stability**

The stability was assessed by LQC, MQC, and HQC samples stored at room temperature up to 3 days.

**4.6. Data analysis**

Pharmacokinetic analyses were performed using Shimadzu LabSolutions, and pharmacokinetic parameters were calculated using a non-compartmental model in DAS 2.0 software (BioGuider, China). Concentration–time profile was further generated using GraphPad Prism 10.1.2.
